# Enteral nutrition for severe malnutrition Crohn's disease patient: Case report

**DOI:** 10.1097/MD.0000000000020112

**Published:** 2020-06-19

**Authors:** Xuemei Li, Qian You, Yang Song, Lei Shi, Wen Hu

**Affiliations:** a Clinical Nutrition Department, West China Hospital of Sichuan University; b Sichuan Center for Di0sease Control and Prevention, Chengdu, China.

**Keywords:** crohn's disease, enteral nutrition, malnutrition

## Abstract

**Introduction:**

Crohn's disease (CD) is a chronic systemic inflammatory disease with indefinite remission and relapse cycles, which can result in a high incidence rate of malnutrition. There has been increasing clinical interest in enteral nutrition (EN) as an adjunct treatment for CD. This report aims to present a case of a severely malnourished CD patient given EN support in combination with conventional infliximab (IFX) treatment.

**Patient concerns:**

A 42-year-old CD patient with severe malnutrition. She once weighted 27.5 kg (BMI 11.4 kg/m^2^) with hyponatremia, which may cause sudden death at any time.

**Diagnosis:**

In this study, the diagnosis of CD made by our gastroenterologist was based on integrating patient symptoms, radiologic findings, and biopsy results. The patient had no differential diagnosis.

**Interventions:**

The patient received EN support and actively followed up for more than 2 years. The patient also received IFX treatment and four surgeries on an as-needed basis to manage her symptoms.

**Outcomes:**

The patient's inflammation and symptoms were finally improved by a combination of enteral nutrition and IFX, and her body weight increased to 44 kg.

**Conclusion:**

The jejunal feeding tube was the starting point of her weight gain and inflammation reduction, which allowed her adequate energy. EN may be a potential complementary therapeutic strategy to manage clinical symptoms of CD and improve severe malnutrition.

## 1 Introduction

Crohn's disease (CD) is a condition with chronic and recurrent intestinal inflammation mediated by abnormal immunity. It can be caused by a variety of factors and tends to recur for life. In recent years, the incidence of CD has been increasing worldwide. This phenomenon first appeared in North America and Nordic Europe, where the social economy is highly developed. In the past, the disease was extremely rare in China, but it has now become a common disease in China.^[1]^

CD has cycles of remission and relapses that can occur for life, and it often requires expensive and demanding long-term treatments. A cohort study showed that traditional treatment failed to change the natural course and outcomes of CD.^[2]^ A third of CD patients worldwide are still hormone dependent.^[3]^ At least 50% of patients need surgical treatment within the first 10 years of the disease, and the recurrence rate is as high as 45% to 55%.^[4]^ Twenty percent of CD patients need to be hospitalized every year due to worsening of condition, and the long-term remission rate is only 10%.^[5]^ Additionally, indirect burdens to CD patients, such as fragility or loss of the ability to work, cannot be ignored. One study showed that about 75% of patients were unable to take full-time jobs at the time of diagnosis, and 15% of the patients lost their ability to work after 5 to 10 years of illness.^[6]^ Furthermore, since the high-risk populations of CD are young and middle age, this undoubtedly produces a huge economic burden to society as well.

However, the pathogenesis of CD is still unclear. Currently, the potential pathogenesis of CD is proposed to be caused by the interactions of genetic factors, environmental factors (diet, smoking, hygienic conditions, lifestyle, etc.), intestinal bacteria, and intestinal immune system disorders. There is a hypothesis that an ordinary diet contains harmful ingredients that can lead to inflammation. Therefore, total parenteral nutrition (TPN) was initially proposed for active CD patients to allow “full rest” of the diseased intestinal segments.^[7]^ However, there was no significant difference in remission rates between parenteral nutrition (PN) and enteral nutrition (EN) in patients with active CD.^[8]^ More importantly, after TPN treatment, the intestinal tract lost mechanical stimulation. This leads to intestinal mucosal atrophy and thinning and intestinal barrier dysfunction, which may aggravate the inflammatory response of CD.^[9]^ The essential EN formula can reduce the inflammatory ingredients in the diet, and it can also maintain intestinal function and prevent mucosal atrophy. Therefore, in addition to aminosalicylic acid, hormones, azathioprine, and biological agents commonly used in the clinical setting, EN could provide new insights into the clinical treatment of active CD.

Here, we describe a case of a female CD patient with severe malnutrition that has gone through tremendous difficulties and desperation during her course of treatment. This article was written according to CARE guidelines, and the patient agreed to sign the informed consent to publish the case.

## 2 Case report

### 2.1 The first hospitalization

A 42-year-old female patient was admitted to our hospital on September 18, 2015. She had a 4-year history of recurrent oral ulcer, a 1-year history of right abdominal pain, a 6-month history of perineal ulcer, and a 4-month history of abdominal mass. She was diagnosed with an appendiceal abscess with cecum rupture and perforation in 2014 at a local county hospital. She received a partial cecum ileocecal resection and intestinal anastomosis. Then, an abscess incision and drainage were performed twice at that hospital. Fecal fluid discharge was observed during each procedure. Six months prior, she underwent an excision of a perineal ulcer at the local county hospital.

A CT scan of the intestinal tract at our hospital indicated that her CD was multi-series. There were perforation of intestines and formation of a local abscess near the original surgical incision on the anterior abdominal wall. Her colonoscopy showed polypoid change. The pathologic biopsy indicated severe chronic active inflammation of mucosa.

Laboratory tests revealed that HB was 99 g/L, ALB was 30.1 g/L, and ESR was 113 mm/h. Her admission diagnosis was CD with possible intestinal fistula.

On October 13, 2015, she underwent several surgical procedures at our hospital. These procedures included exploratory laparotomy, resection of the diseased small intestine, ileum end ostomy, and right lower abdominal sinus clean-up. She also received nutritional therapy during the perioperative period, which will be discussed below. Due to her poor economic status, 1 month after surgery, she had to choose azathioprine instead of infliximab (IFX).

This patient had a weight loss of 15 kg prior to this hospitalization. Her admission body weight was 34 kg and her body mass index (BMI) was 14.2 kg/m^2^. Our gastrointestinal surgeons removed her diseased small intestine and made a temporary ileostomy. This was done to remove the irritation of antigens from food and to optimize the “full rest” of the lower intestinal tract. It is worth mentioning that, even at this life-threatening point, the patient refused to use IFX and long-term enteral nutritional support for economic reasons. This laid the basis for the twists and turns of the rest of her treatment process.

### 2.2 The second hospitalization

On January 21, 2016, the patient presented to our hospital again and complained of CD (penetrating non-stenotic enterocolic active phase), ileostomy, and severe malnutrition. She was prescribed levofloxacin and fluconazole for anti-infection, diarrhea control, intestinal flora regulation, EN, and PN. However, pneumothorax occurred when the subclavian vein catheterization was performed, and a thoracic vent was placed. As the extra air gradually leaked out, the vent was pulled out. From her abdomen, yellow water-like stool was drained from the ileostomy.

At the second admission, the patient's weight had decreased to 27.5 kg (BMI 11.4 kg/m^2^). She also presented with hyponatremia, which could lead to sudden death at any time. There were multiple reasons for her severe malnutrition and hyponatremia. The primary reason was the considerable loss of electrolytes and water. The average drainage from her ileostomy was 500 and 1300 mL/day. In the early stage of post-operation, the daily excretion was as high as 1800 mL. When discharged, the patient was not well instructed on how to replenish enough water and electrolytes at home. The second reason was that the patient had severe dietary restrictions to prevent an ileostomy blockage caused by food. A fecal stone can form if the patient consumed food with insoluble fiber, which may block the proximal end of the foramen. This also resulted in a fear of eating, which deteriorated her nutritional status. This particular patient was severely malnourished and weighed only 27.5 kg. Thus, improving her nutritional status should be an integral part of treatment. However, PN administration failed because of pneumothorax. Therefore, in this second hospitalization, the patient only received drug therapy with azathioprine and cholestyramine, but she was still severely malnourished when discharged.

### 2.3 The third hospitalization

**Figure 1:**
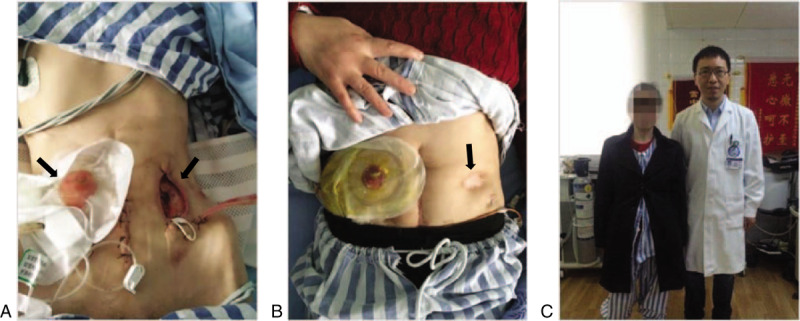
The picture (A) showing that during the 3rd hospitalization after the patient received the second small intestinal stoma surgery with weight of 28 kg (the new small intestinal stoma was the left arrowhead, the old small intestinal stoma was the right arrowhead); the picture (B) showing that the patient's old small intestinal stoma was going to heal (the right arrowhead) with the obvious weight gain; the picture (C) showing that the patient received operation of small intestinal stoma closure with weight of 44 kg (the person on the right was Shi Lei, one of our authors and the patient's clinical dietitian in charge).

On May 31, 2016, the patient was admitted again because of a fistula rupture. At great risk, our gastrointestinal surgeon completed an operation consisting of small intestinal stoma closure, enterocolic anastomosis, enterostomy, jejunal nutrition tube implantation, and intestinal adhesion lysis on June 6, 2016. Because of her fistula rupture, the surgeons had to perform the small intestinal stoma closure, and they made a new small intestinal stoma (Fig. 1A). The jejunal nutrition tube provides another access for nutrition intake in which enteral nutrition could be injected. Normal food could still be ingested orally and then turned into food residue, which is discharged from the upper fistula bag. Therefore, after the third hospitalization, the patient gained two routes for feeding, oral and jejunal. This boosted her energy and also reduced exposure to antigens in the remaining vulnerable intestine. This time, she finally received IFX as her treatment option. On March 30, 2017, the patient's former fistula completely healed (Fig. 1B).

### 2.4 The fourth hospitalization

Two years later, on March 20, 2018, the patient came back to receive the stoma closure surgery. Her physicians and surgeons were surprised to see that she weighted 44 kg this time (Fig. 1C). We performed the operation on April 2, 2018, and she ran a fever because of an abscess on the abdominal wall. Thus, she was then prescribed anti-infective medication after the incision, and it was continued through the IFX treatment. There are several questions left to be answered about this change in the patient's status. What allowed her to gain so much weight, which allowed her to undergo the stoma closure surgery? What had changed in the past 2 years? Was it the selection of IFX or the administration of nutrition therapy? What played the most important role?

### 2.5 The nutrition therapy

According to the ESPEN guideline of clinical nutrition in inflammatory bowel disease,^[10]^ the energy requirements of patients with IBD are similar to those of the healthy population. However, the protein requirements are increased to 1.2 to 1.5 g/kg/day in adults with active IBD, which is higher than that of the general population. Therefore, the initial goal of nutritional therapy for this patient was set at 1500 kcal and 60 to 75 g protein. However, in the process of achieving this goal, the nutrition plan had undergone 8 adjustments. The details of the nutrition plan are shown in Table 1. During the second hospitalization, the patient received nutritional therapy only while in the hospital, which is a short period of time. A pneumothorax occurred when the subclavian vein catheterization was performed. We thought that the pneumothorax was related to the very low BMI of the patient. Fortunately, she continued the enteral nutrition after the second and third hospitalizations.

**Table 1:**
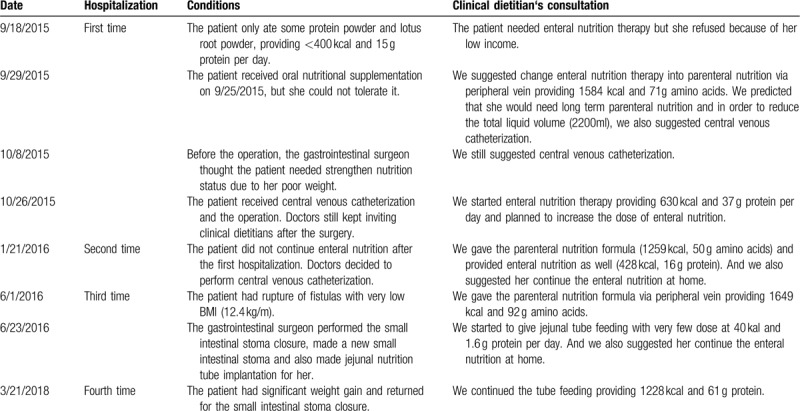
Timeline of the patient's clinical course and changes of nutrition therapy plan.

A review and analyzation of the entire nutritional therapy for the patient, including the duration and details, is discussed below. Home enteral nutrition lasted for 2 years and 3 months, which accounted for a very large proportion of her total treatment course. This also happened to be the period of time in which she gained weight. Interestingly, we had unintentionally tried all methods of nutritional therapy, including PN via peripheral vein, PN via central venous catheterization, oral nutritional supplementation, nasogastric feeding tube, and jejunal feeding tube. The jejunal feeding tube lasted for 664 days, and it played a very important role in her treatment, providing 1200 to 1500 kcal/day. A significant reduction in inflammatory markers and an increase in body weight (Table 2) was observed after 9 months of EN. However, we noticed an increase in her triglycerides at the fourth hospitalization.

**Table 2:**
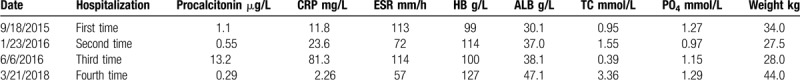
Changes of the patient's laboratory tests during the treatment.

## 3 Discussion

When the patient's triglyceride increased, she did not receive any PN or glucocorticoid. We suspected that a high ratio of carbohydrates caused the increase in triglycerides. The initial percentage of carbohydrates was 60%, and it increased to 64%, which lasted for more than 2 years. After we reduced the portion of carbohydrates to 52%, the triglycerides were reduced to 2.08 mmol/L.

We attribute her successful treatment to multiple reasons. First, her remaining small intestine was more than 200 cm, which could guarantee an area for essential absorption. In the first operation, some ileum and cecum were cut, so the vitamin B_12_ level could have been affected. The second operation was an ileostomy, so the absorption of potassium, short-chain fatty acids, and vitamin K could have been affected. In the third operation, ulceration had formed at the original stoma site. Thus, a new stoma was established, and a jejunal nutrition tube was placed. The start of the jejunal tube feeding was the key point of her weight gain. It was reported that jejunal feeding of intact casein resulted in more rapid protein digestion and amino acid absorption, compared to gastric feeding.^[11]^ A greater postprandial increase in circulating essential amino acid concentrations may allow for a more robust increase in the muscle protein synthesis rate after jejunal casein feeding. The jejunal feeding tube reduced the patient's inflammation, while providing adequate energy in a socially acceptable way.

The second reason for the patient's success was that the patient accepted IFX and home enteral nutrition, and she completed her treatment despite her poor economic condition. Most impressively, her loving husband visited our outpatient nutrition service every month to take the enteral nutrition formula home for her on regular basis.

Thirdly, the enteral nutrition we prescribed provided her with more than 1200 kcal/day, which may have also played a key role in the success of the treatment. Research has suggested that when patients with CD were treated with scheduled maintenance IFX treatment, concomitant EN treatment ≥600 kcal/day reduced the incidence rate of loss of response to IFX.^[12]^ It also confirmed that the type of EN formula (elemental and/or polymeric formulas) did not affect the results.

Most importantly, the patient adhered to the treatment for more than 2 years, and she actively followed up with a clinical dietitian. This was a classic case of home enteral nutrition management, which is the continuation of a nutritional intervention in the hospital. Up to the time of writing this case report, the patient has achieved a body weight of 55 kg. Finally, we conclude that home enteral nutrition cannot only help to treat diseases, but it can also give patients a sense of safety. From the patients’ perspective, they can always trust and rely on clinical dietitians, to who give continuous help and support that can be both physiological and spiritual.

## Author contributions

**Conceptualization:** Lei Shi.

**Investigation:** Xuemei Li.

**Resources:** Lei Shi.

**Validation:** Wen Hu.

**Visualization:** Xuemei Li.

**Writing – original draft:** Xuemei Li.

**Writing – review & editing:** Lei Shi, Qian You, Wen Hu.
